# Clinical performance testing of the automated haematology analyzer XN-31 prototype using whole blood samples from patients with imported malaria in Japan

**DOI:** 10.1186/s12936-022-04247-x

**Published:** 2022-07-30

**Authors:** Kanako Komaki-Yasuda, Satoshi Kutsuna, Miki Kawaguchi, Mina Kamei, Kinya Uchihashi, Keiji Nakamura, Takato Nakamoto, Norio Ohmagari, Shigeyuki Kano

**Affiliations:** 1 Department of Tropical Medicine and Malaria, Research Institute, National Center for Global Health and Medicine, 1-21-1 Toyama, Shinjuku-ku, Tokyo, 162-8655 Japan; 2 Sysmex Corporation, 4-4-4 Takatsukadai, Nishi-ku, Kobe, 651-2271 Japan; 3 Disease Control and Prevention Center of National Center for Global Health and Medicine, 1-21-1 Toyama, Shinjuku-ku, Tokyo, 162-8655 Japan

**Keywords:** Automated hematology analyzer, Flow cytometry, XN-31p, Diagnosis, Malaria

## Abstract

**Background:**

The automated haematology analyzer XN-31 prototype (XN-31p) is a new flow cytometry-based device developed to measure the number and the ratio of malaria-infected red blood cells (MI-RBC) with a complete blood count (CBC). The XN-31p can provide results in about one minute and also can simultaneously provide information on the malaria parasite (*Plasmodium*) species. In this study, clinical testing of the XN-31p was performed using blood samples from patients with imported malaria in Japan.

**Methods:**

Blood samples were collected from 80 patients who visited the hospital of the National Center for Global Health and Medicine, Tokyo, Japan, for malaria diagnosis from January 2017 to January 2019. The test results by the XN-31p were compared with those by other standard methods, such as microscopic observation, rapid diagnostic tests and the nested PCR.

**Results:**

Thirty-three patients were diagnosed by the nested PCR as being malaria positive (28 *Plasmodium falciparum*, 2 *Plasmodium vivax*, 1 *Plasmodium knowlesi*, 1 mixed infection of *P. falciparum* and *Plasmodium malariae*, and 1 mixed infection of *P. falciparum* and *Plasmodium ovale*), and the other 47 were negative. The XN-31p detected 32 patients as “MI-RBC positive”, which almost matched the results by the nested PCR and, in fact, completely matched with the microscopic observations. The ratio of RBCs infected with malaria parasites as determined by the XN-31p showed a high correlation coefficient of more than 0.99 with the parasitaemia counted under microscopic observation. The XN-31p can analyse the size and nucleic acid contents of each cell, and the results were visualized on a two-dimensional cytogram termed the “M scattergram”. Information on species and developmental stages of the parasites could also be predicted from the patterns visualized in the M scattergrams. The XN-31p showed a positive coincidence rate of 0.848 with the nested PCR in discriminating *P. falciparum* from the other species.

**Conclusions:**

The XN-31p could rapidly provide instructive information on the ratio of MI-RBC and the infecting *Plasmodium* species. It was regarded to be of great help for the clinical diagnosis of malaria.

**Supplementary Information:**

The online version contains supplementary material available at 10.1186/s12936-022-04247-x.

## Background

The species of *Plasmodium* that cause human malaria include *Plasmodium falciparum*, *Plasmodium vivax*, *Plasmodium ovale*, and *Plasmodium malariae*. In addition, *Plasmodium knowlesi*, one of the causative pathogens of malaria in monkeys, sometimes infects human and causes severe malaria symptoms in Southeast Asian regions [1–3]. Rapid and accurate detection of *Plasmodium* species in a patient’s blood can lead to adequate treatment of malaria. In particular, discrimination of *P. falciparum* from other species is very important, since patients with *P. falciparum* can rapidly develop high parasitaemia leading to unexpected lethal outcomes.

The gold standard in the diagnosis of malaria is detection of parasites by microscopic observation of Giemsa-stained blood smears [4]. The ratio of *Plasmodium* infected red blood cells (RBCs) can be calculated, and indeed, *Plasmodium* species can be identified according to their morphological characteristics. However, the sensitivity and specificity of the diagnosis depend on the skill of the microscopists.

Additional differential diagnostic techniques are currently available. Rapid diagnostic tests (RDTs) are immunochromatographic test kits designed to detect malaria parasite-specific antigens in the patient’s blood qualitatively [4]. The RDTs use simple methods and require only about 15 min to obtain results. However, the sensitivity and specificity of the RDTs are relatively low in comparison with other diagnostic methods. In addition, mutations in the HRP-2 antigen are rapidly spreading, which are causing false negative results of the RDTs [5]. Contrastingly, PCR-based diagnostic methods are highly sensitive and can differentiate *Plasmodium* species using the different genetic sequences of parasite DNAs [6–8]. However, PCR also requires skilled laboratory techniques, and it takes 3 to 6 h to obtain the qualitative results.

To solve those challenges, the automated haematology analyzers XN-31p were developed based on the principle of flow cytometry by the Sysmex Corporation (Kobe, Japan) to detect malaria parasites in patient’s blood. Information on MI-RBC is suggested by the XN-31p in about 1 min after placing a whole blood sample in the system without any special pre-treatment of the sample.

As a matter of fact, the XN-30 was first released for research use only, and the accuracy and reliability of its counting parasite numbers of *P. falciparum* in blood samples were well confirmed at some clinical sites in malaria-endemic areas [9, 10]. Then, functionally identical XN-31p was developed for in vitro diagnostic methods. The XN-31p means a prototype device before the release of the XN-31 commercially which has the same physical characteristics, hardware configuration, and reagent composition as does the XN-31p. For the XN-31p and the XN-31, their excellent performance as malaria diagnostics in clinical settings were also suggested in recent reports respectively [11–13].

In this study, clinical testing of the XN-31p for the detection of malaria parasites in patient blood was performed to evaluate the confidence of the data in comparison with the results provided by the standard diagnostic methods of the RDT, microscopic observation, and PCR in a clinical setting using patients with suspected imported malaria in Japan.

## Methods

### Patients

Eighty-six suspected patients who visited the Center Hospital of the National Center for Global Health and Medicine (NCGM) from January 2017 to January 2019 were asked to be subjects in the clinical testing of the XN-31p. Of them, 82 were enrolled, and the other 4 patients did not give their consent to this study.

Of the 82 registered cases, 1 case that was found to be incompatible with the selection criteria (the patient did not have a travel history within 1 year of the consulting the hospital) was dropped. In addition, another case whose measurement results could not be obtained within 24 h of blood collection was excluded from the analysis. Finally, 80 cases were included and monitored through their treatment procedure by collecting daily EDTA-preserved venous blood samples.

### The automated haematology analyzer XN-31p

The automated haematology analyzer XN-31p (Sysmex Corporation) can detect malaria parasite-infected RBCs, CBC based on the principle of flow cytometry. The XN-31p automatically counts and classifies cells by irradiating cells with a 405-nm laser beam and analysing the resultant forward-scattered light (FSC) and side fluorescent light (SFL). In the XN-31p, the blood cells are initially treated automatically with Lysercell M and Fluorocell M (Sysmex Corporation). Lysercell M shrinks the RBCs, and the size of these shrunken cells well reflects the parasite cell size within the RBCs [14]. Then, treatment with the Fluorocell M stains the nucleic acids inside the cells. After these treatments, the *Plasmodium* infected RBCs are effectively analysed according to the intensity of the FSC and SFL of each cell. The intensity of the FSC signal mainly reflects the size of the cells, whereas that of the SFL reflects the amount of each cell’s nucleic acids. These signals are consequently used to detect malaria-infected RBCs (MI-RBC) with the help of unique algorithms.

This study measured patient blood samples by two modes of the XN-31p: the LM (low malaria) mode and the PD (pre-dilution) mode. The LM mode uses whole blood samples and was optimized to count low numbers of *Plasmodium* infected RBCs. The PD mode uses blood samples diluted 7 times with CELLPACK DCL (Sysmex Corporation). Sample of 60 µL was used for measurement for the LM mode and 70 µL was used for the PD mode. The blood samples were measured by both modes of the XN-31p within 24 h of blood collection from patients. For the PD mode, the samples were measured immediately after the blood dilution.

Information on the species of malaria parasites can be suggested by flagging performed by the haematology analyzer algorithm. Namely, “Malaria? (P.f)” is the flagging suggesting *P. falciparum*, and “Malaria? (Others)” is the flagging suggesting *Plasmodium* species other than *P. falciparum*. When the malaria parasite is unable to be classified, flagging of “Malaria? (UNC)”, meaning unclassified parasites, is suggested. Moreover, in rare instances, the detection of unknown particles in the blood sample sometimes causes indeterminant results, and then, flagging of “MI-RBC Abn Scattergram” is suggested, which means abnormal scattergram.

### Microscopic observation

Thin blood smears were prepared with the patient’s blood. After fixation with methanol, the blood smears were stained with Giemsa solution. Ratios of *Plasmodium* infected RBCs were calculated based on visual counting under microscopic observation. Gametocytes were also included in the parasite density if any. The counting could be stopped if 50 or more infected RBCs were found when 5000 RBCs were observed (parasitaemia > 1%). In the case that not less than 10 infected RBCs were found when 10,000 RBCs were observed, the counting could also be stopped (parasitaemia > 0.1%). Then, no more than 100,000 RBCs were observed until at least 10 infected RBCs were found (parasitaemia: 0.1–0.01%). The counting was to be terminated when 100,000 red blood cells were observed regardless of the number of the infected RBCs detected (parasitaemia < 0.01%). Thus, samples were declared negative after having observed 0 parasite per 100,000 RBCs. The counting manner is illustrated as a flowchart in Additional file 1.

### Rapid diagnostic test

For the RDT, BinaxNOW® Malaria (Binax, Inc., Scarborough, ME, USA) was used according to the manufacturer’s procedure. The T1 line is formed when *P. falciparum*-specific histidine-rich protein 2 antigen is detected. The T2 line appears when aldolase, a pan-*Plasmodium* antigen, is captured. Namely, the T1(+)T2(+) result can be interpreted as a single infection with *P. falciparum* or combined infection with *P. falciparum* and other *Plasmodium* species, T1(+)T2(−) as a single infection with *P. falciparum*, T1(−)T2(+) as an infection with non-falciparum *Plasmodium* species, and T1(−)T2(−) as negative for malaria infection.

### PCR

The DNA samples were extracted from fresh or frozen patient blood, and 200 µL of blood was used for the extraction. DNA extraction was accomplished with a Maxwell DNA RSC kit (Promega, Madison, WI, USA) or Qia DNA mini kit (Qiagen, Venlo, The Netherlands). The nested PCR targeting the 18SrRNA gene of 4 human malaria parasite species and *P. knowlesi* was performed to verify the *Plasmodium* species [8].

## Results

### Overview of clinical samples used in this study

**Fig. 1:**
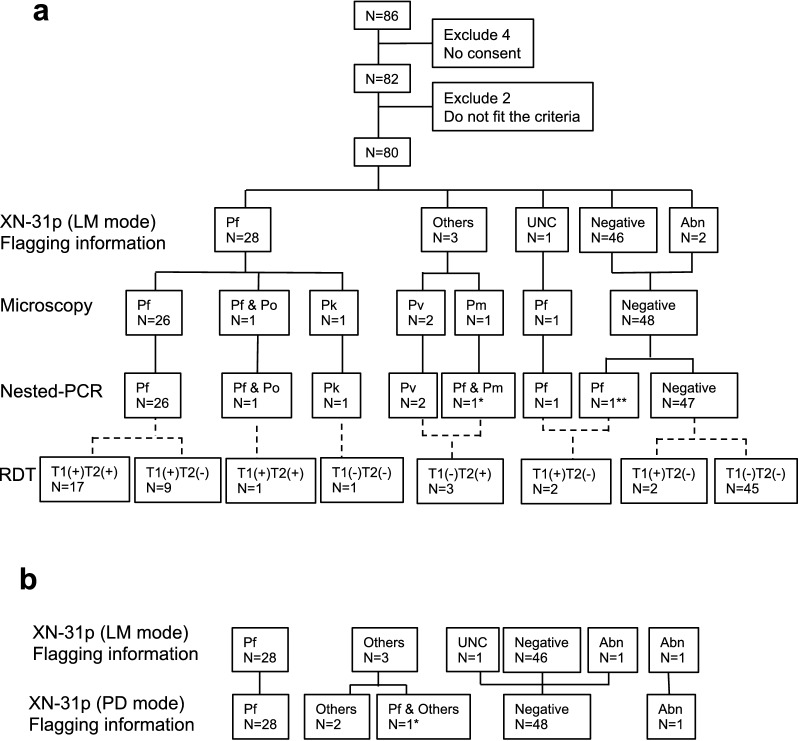
Flow diagram of the malaria diagnostic tests in this study. **a** Results of the different diagnostic tests such as the LM mode (mode for whole blood samples) of the XN-31p, microscopy, and the nested PCR are shown. Results of RDTs were indicated in the bottom column. **b** Comparison of the test results by the 2 different modes of the XN-31p. For the results of XN-31p, Pf, Others, UNC and Abn indicate the flagging information of “Malaria? (P.f)”, “Malaria? (Others)”, “Malaria? (UNC)” and “MI-RBC Abn Scattergram”, respectively. Pf: *P. falciparum*, Pv: *P. vivax*, Po: *P. ovale*, Pm: *P. malariae,* Pk: *P. knowlesi.* *This sample was proved to be *P. malariae* infection by the microscopy and *P. malariae*–*P. falciparum* mixed infection by the nested PCR. **Samples showing discrepant results between microscopy and the nested PCR

A flow diagram of all patients recruited to this study is shown in Fig. 1, and a detailed profile of the patients is listed in Additional file 2. All 80 patients who were included in the analysis had a history of travel to malaria-endemic areas during the 1 year before visiting the NCGM hospital. The places travelled to included countries in Africa (61 cases), South America (4 cases), Asia (3 cases), and the West-Pacific regions (14 cases). In fact, 28 cases from Africa were positive by PCR.

### Comparison of test results by XN-31p analysis and other methods

Results obtained with the different diagnostic methods are summarized in Fig. 1. With the LM mode of the XN-31p, 28 samples were detected as *P. falciparum* positive by the flagging message of “Malaria? (P.f)” and 3 received the message of “Malaria? (Others)” (Fig. 1a). One sample showing the flagging message of “Malaria?(UNC)” was proved to be *P. falciparum* positive by microscopy, the nested PCR, and RDT, suggesting that the *Plasmodium* species was not perfectly classified by the XN-31p. With the LM mode, 46 samples were detected as negative, and 2 were shown to have inconclusive results by the flagging message of “MI-RBC Abn Scattergram”. The 2 samples, which were detected to be “MI-RBC Abn Scattergram” by the LM mode, were verified to be negative by microscopy, the nested PCR, and RDT.

Of the 28 samples detected as *P. falciparum* positive by the LM mode of the XN-31p, 26 samples were also diagnosed as *P. falciparum* infection by microscopic observation. The other 2 samples were detected by microscopic observation to be mixed infection of *P. falciparum* and *P. ovale* and *P. knowlesi*, infection, respectively. Of the 3 samples flagged with “Malaria? (Others)” by the LM mode, 2 samples were confirmed to be *P. vivax* infections, and the other 1 sample was proved to be *P. malariae* infection by microscopy.

The results of microscopic observations were well concordant with the nested PCR, except for 1 negative by microscopy that was, in fact, *P. falciparum* positive by the PCR, and 1 *P. malariae* positive by microscopy that was revealed to be a *P. falciparum* and *P. malariae* mixed infection by the PCR. In this *P. falciparum* and *P. malariae* mixed infection, the specific band for the PCR product made with the *P. falciparum* -specific primers on gel electrophoresis was very pale (data not shown), and the RDT result was T1(−)T2(+), suggesting that the density of *P. falciparum* parasites in the peripheral blood of the patient was low enough to be at submicroscopic level.

The results with the PD mode of the XN-31p were almost the same as those with the LM mode except for 3 samples (Fig. 1b). Of them, 2 samples were flagged with “Malaria? (UNC)” and “MI-RBC Abn Scattergram” respectively by the LM mode but both were negatives by the PD mode. The other sample was flagged with both “Malaria? (P.f)” and “Malaria? (Others)” by the PD mode but with only “Malaria? (Others)” by the LM mode.

### Evaluation of XN-31p efficiency

The sensitivity, specificity, positive predictive value, and negative predictive value for the ability of each method to detect malaria parasites were calculated in comparison with the results of the nested PCR as the standard method (Table 1). The LM mode of the XN-31p showed the same sensitivity as that of microscopic observation, but the sensitivity of the PD mode was slightly lower. The LM mode of the XN-31p showed the same sensitivity as that of microscopic observation, but the sensitivity of the PD mode was slightly lower. The specificities of microscopy and both XN-31p modes were 100%. Efficiency can be calculated by taking the positive and negative results divided by the total included sample number, and then, 98% for the LM mode and 99% for the PD mode. The ability of RDT to detect malaria parasites was evaluated based on a combination of the results of the T1 and T2 bands. The specificity of the RDT was lower than those of the LM and PD modes of the XN-31p. The sensitivity of the T1 band for *P. falciparum* detection ability was almost the same as that of microscopy, but the specificity was lower. The sensitivity of the T2 band for pan-malaria parasite detection was very low, but the specificity was 100%.

**Table 1:** Sensitivity, specificity, PPV and NPV of each diagnostic method

Method	Sensitivity (%)	Specificity (%)	PPV (%)	NPV (%)
Microscopy	97	100	100	98
XN-31p LM mode^a^	97	100	100	98
XN-31p PD mode^a^	94	100	100	96
RDT	97	96	94	98
RDT T1^b^	97	96	94	98
RDT T2^c^	64	100	100	80

All values were calculated using the results of the nested PCR as a standard

^a^Some cases flagged as “MI-RBC Abn” in were excluded from the calculation

^b^The values regarding the *P. falciparum* detection ability of the T1 band were calculated

^c^The values regarding the Pan-parasites detection ability of the T2 band were calculated

The abilities of microscopy and the XN-31p to discriminate *Plasmodium* species were compared with that of the nested PCR, and the discrimination ability between *P. falciparum* infection, non-*P. falciparum* (others) infection, and *P. falciparum* and other mixed infections was evaluated. The coincidence rates were calculated using the results of 33 PCR-positive samples. The LM and PD modes of the XN-31p showed a coincidence rate of 0.848 and 0.906 respectively, which were lower than that of 0.938 by microscopy (detailed data are shown in Additional file 3). *Plasmodium knowlesi* infection detected by microscopy was detected as “Malaria? (P.f)” by both modes of the XN-31p, and *P. falciparum* and *P. ovale* mixed infection detected by microscopy was detected as “Malaria? (P.f)” by the LM and PD modes of the XN-31p (see Fig. 1).

### Simultaneous reproducibility of parasite counting ability

Information on the number of *Plasmodium* infected RBCs per µL of blood, and the ratio of *Plasmodium* infected RBCs to total RBCs are given by the XN-31p as “MI-RBC#” and “MI-RBC%”, respectively. Five samples (3 *P. falciparum* samples with different range of parasitaemia and 2 non-*P. falciparum* samples) were chosen and tested in 10 repetitive measurements (see Additional file 4). In both the LM and PD modes, the coefficient of variations (CV%) of MI-RBC# and MI-RBC% were lower than 10% for all samples, indicating that simultaneous reproducibility of the parasite counting ability of the XN-31p was satisfactorily high.

### Quantitative parasite detection ability of the XN-31p versus microscopic observation

**Fig. 2:**
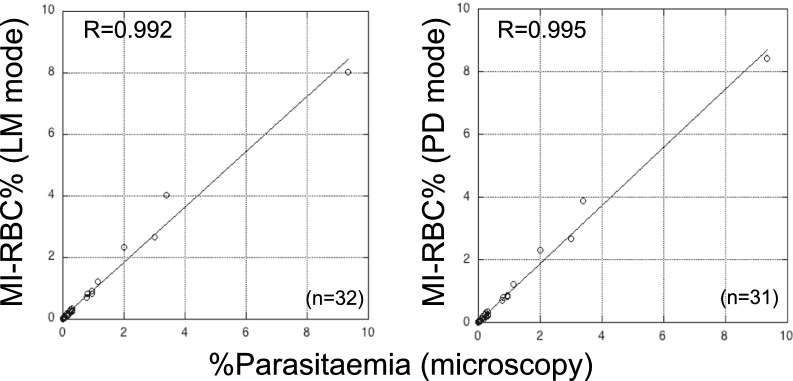
Comparison of %parasitaemia by microscopy and the ratio of *Plasmodium* infected RBCs determined by the XN-31p. The simple linear regression line and the correlation coefficient are indicated inside each column

For the 32 samples positive by microscopy, % parasitaemia was compared with the MI-RBC% value determined by the LM and PD modes of the XN-31p. The values were shown to be highly correlated, as indicated by correlation coefficients of > 0.99 (Fig. 2).

### Typical M scattergram patterns measured by the XN-31p

**Fig. 3:**
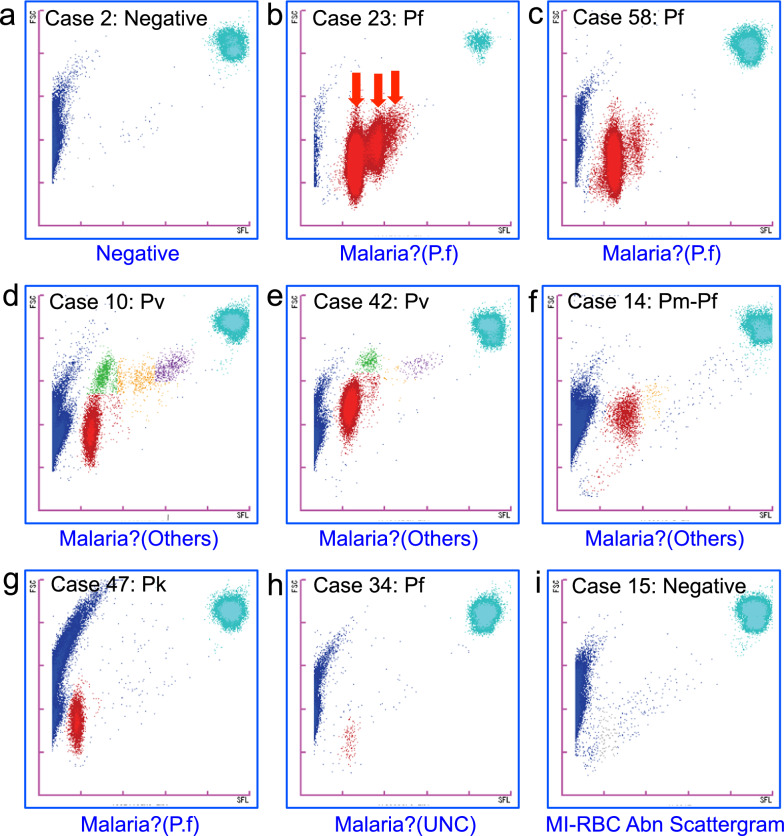
Typical patterns of M scattergrams by the LM mode of the XN-31p. The flagging messages obtained by measurement with the XN-31p are indicated under each M scattergram. Each dot in the M scattergrams represents the x (SFL): y (FSC) co-ordinates of each individual blood cell. **a** Negative case. **b** *P. falciparum* case with high parasitaemia. Each cluster is indicated by a red arrow. **c** *P. falciparum* case with low parasitaemia. **d**, **e** *P. vivax* cases. **f** *P. malariae* and *P. falciparum* mixed infection case. **g ***P. knowlesi* case. **h** *P. falciparum* case by microscopy that was flagged as “Malaria?(UNC)”.** i** Negative case that was flagged as “Abn”

The XN-31p can analyse the characteristics of the blood cells according to the FSC and SFL information of each cell and then indicate the distribution of cells as M scattergrams. Each developmental stage of the parasites is classified automatically and presented as a coloured cluster. Typical patterns of the M scattergrams obtained by the LM mode are shown in Fig. 3. For negative cases of malaria, uninfected normal RBCs are indicated as blue clusters at the left side of the M scattergram, and white blood cells are indicated as a light blue cluster in the upper right area of the scattergram (Fig. 3a).

Figure 3 shows typical M scattergram patterns for *P. falciparum* infections of high parasitaemia (9.32%, Fig. 3b) and low parasitaemia (0.25%, Fig. 3c). In these M scattergrams, only the ring forms were detected and shown as red clusters. For the high-parasitaemia case, RBCs infected with 1, 2, or 3 ring forms were indicated as clusters with different intensities of SFL in the M scattergram, suggesting that different amounts of cellular nucleic acids were reflected. In both cases, the XN-31p provided information about the *Plasmodium* species by flagging “Malaria?(P.f)”.

In the slide smears of 2 *P. vivax* cases, RBCs infected with malaria parasites of various growth stages such as ring form, trophozoite, schizont, and gametocyte were found by microscopy. Those parasites were considered to correspond to each coloured cluster shown in the M scattergrams (Fig. 3d, e). The ring form cluster (red) of *P. vivax* appeared at a position where the FSC intensity was slightly higher than that of *P. falciparum*, and various coloured clusters indicating each growth stage were detected. The distributions of the developmental stages were different by the cases, and the patterns were well concordant with the images obtained by microscopic observations. The flagging messages for the *Plasmodium* species by the XN-31p were “Malaria?(Others)”.

One *P. malariae* case was detected microscopically (Additional file 2, case no. 14). In fact, this case was proved to be a mixed infection of *P. malariae* and *P. falciparum* by the nested PCR, suggesting that the number of *P. falciparum*-infected RBCs was under the detection limit by microscopy. Parasitaemia by microscopy was 0.021% and showed a large number of trophozoites and some gametocytes of *P. malariae*. They were considered to be all contained in the red-coloured cluster showing higher FSC intensity than that of *P. falciparum* in somewhat of a diffuse shape (Fig. 3f). The flagging by the XN-31p was “Malaria?(Others)”.

One *P. knowlesi* case with 0.062% parasitaemia was included in this study. Morphologically, the ring forms and trophozoites were difficult to distinguished from those of *P. malariae*. In the M scattergram, the ring form cluster (red) appeared at a position where the FSC intensity was low (Fig. 3g). Therefore, the flagging message of “Malaria? (P.f)” was given by the XN-31p. However, the cluster appeared at a position of lower SFL intensity as compared with that of the ring form cluster of *P. falciparum* (see Fig. 3b, c).

One case was flagged as unclassified “Malaria? (UNC)” by the LM mode of the XN-31p. In this case, a quite low number of *P. falciparum*-infected RBCs was detected by microscopy (1 parasite per 360,000 RBCs), and the PCR result indicated *P. falciparum* infection. A pale but obvious cluster was detected in the M scattergram (Fig. 3h). The shape and position of the cluster looked similar to those of *P. falciparum*.

Two cases were flagged as “MI-RBC Abn Scattergram” by the LM mode of the XN-31p. The M scattergram of one case is shown (Fig. 3i). In the centre region of the M scattergram, scattered dots of unknown particles could be found. These dots did not form a clearly shaped cluster. No malaria parasites were detected in this sample by microscopy or PCR.

### M scattergram patterns of a mixed infection measured by the XN-31p

**Fig. 4:**
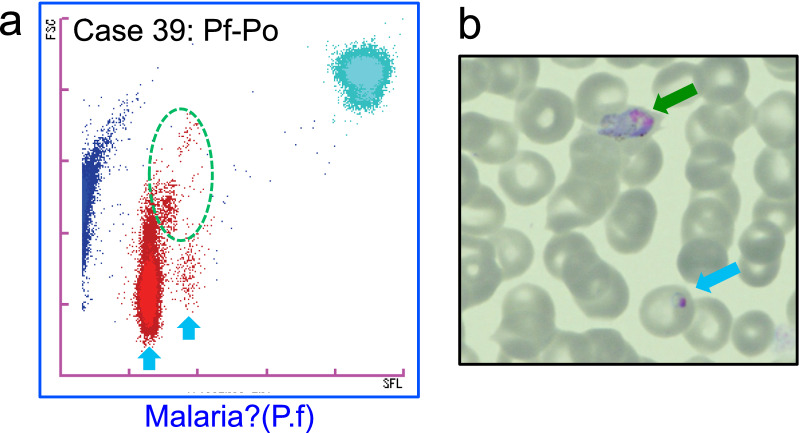
The M scattergrams of a *P. falciparum* and *P. ovale* mixed infection by the LM mode of the XN-31p. **a** The M scattergram of the *P. falciparum* and *P. ovale* infection. Main clusters of *P. falciparum* and a sub-cluster of *P. ovale* are highlighted with blue allows and a green circle, respectively. The flagging messages obtained by measurement with the XN-31p are indicated under the M scattergram.** b** *P. falciparum* (blue arrow) and *P. ovale* (green arrow) were found in the same field of the Giemsa-stained thin blood smear by microscopy

The M scattergram of a case of *P. falciparum* and *P. ovale* mixed infection is shown in Fig. 4a. Typical clusters of *P. falciparum*-infected RBCs were detected. In addition, the presence of non-falciparum parasites was suspected as a small cluster was observed at the upper-right side of the main cluster. In this case, RBCs infected with *P. falciparum* ring forms and *P. ovale* trophozoites were recognized under microscopy (Fig. 4b, 0.1% parasitaemia), of which about 1% of the total *Plasmodium* infected RBCs were *P. ovale* infections. This co-infection was also confirmed by the nested PCR. In this case, the XN-31p gave only the “Malaria? (P.f)” flagging message.

### Detection of non-ring stage parasites in cases of *P. falciparum* infection by the XN-31p

**Fig. 5:**
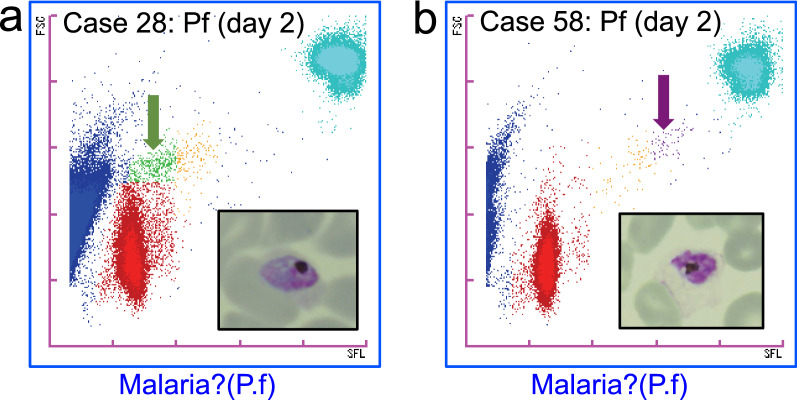
The M scattergrams of *P. falciparum* non-ring stages detected by the LM mode of the XN-31p. The flagging messages obtained by measurement with the XN-31p are indicated under each M scattergram. **a** The M scattergram of the sample from case No. 28 on the second day of illness. *P. falciparum*-infected cells were detected at 0.19% parasitaemia, and the appearance of gametocytes was confirmed by microscopic observation. The gametocytes in the M scattergram are also shown as a green-coloured cluster (indicated by the green arrow). A photo of a gametocyte found in the Giemsa-stained thin blood smear is shown inside the column. **b** The M scattergram of the sample from case No. 58 on the second day of illness. The parasitaemia was 0.047%, and the appearance of schizonts was detected by microscopy. The schizonts in the M scattergram are shown as a purple-coloured cluster (indicated by the purple arrow). A photo of a schizont found in the Giemsa-stained thin blood smear is shown inside the column

In most cases of *P. falciparum* infection, only ring forms can be found in the patient’s peripheral blood because trophozoites and schizonts are usually sequestrated in vascular endothelium [15]. *Plasmodium falciparum* gametocytes can also be found in patients’ peripheral blood, but this is not always the case. This study detected only ring stages in most of the *P. falciparum*-positive cases both by microscopy and the XN-31p, but in several cases, *P. falciparum* gametocytes or schizonts were found under microscopic observation. These non-ring stage *P. falciparum* observations were also detected by the XN-31p and showed specific clusters in the M scattergram (Fig. 5).

### Monitoring of treatment with the XN-31p

**Fig. 6:**
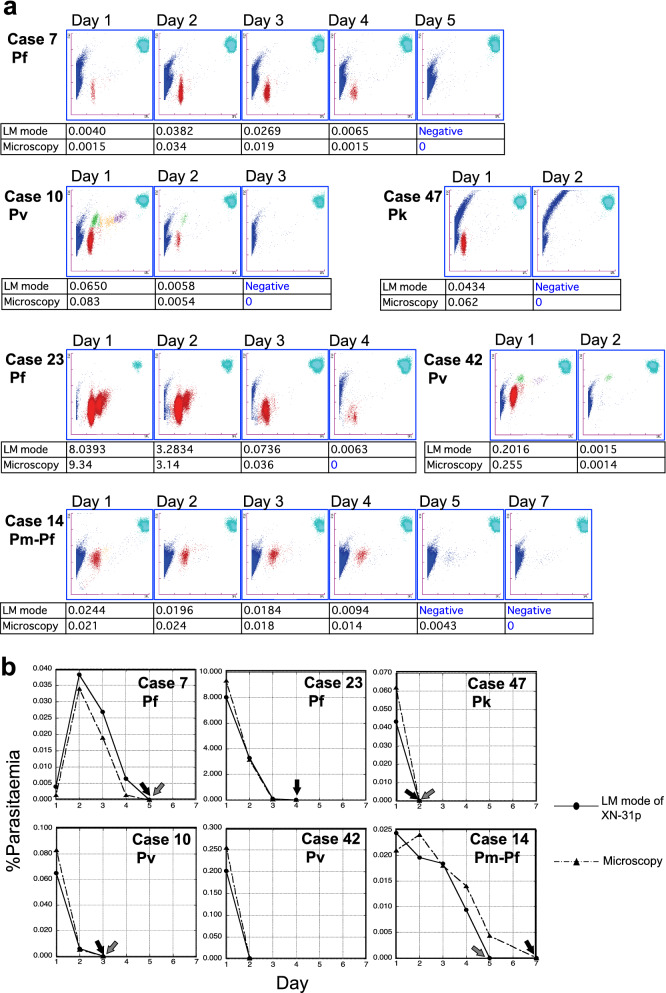
Monitoring of the cases by the XN-31p during the treatment of malaria. **a** The M scattergrams obtained by the LM mode of the XN-31p from daily measurements of the samples of 6 patients during hospitalization. The parasitaemia calculated by microscopy and the MI-RBC% values detected by the LM mode of the XN-31p are shown in the tables under the scattergrams. **b** Changes in parasitaemia in each case during the monitoring period. The horizontal axes show days from the beginning of the treatment, and the vertical axes show parasitaemia detected by microscopy (dashed lines) and LM (solid lines) mode of the XN-31p. The black arrows indicate the days when parasite clearance in the patients’ blood was declared by microscopy, and the gray arrows indicate those detected by the LM mode of the XN-31p. Pf: *P. falciparum*, Pv: *P. vivax*, Pm: *P. malariae*, Pk: *P. knowlesi*

Six patients were monitored during their hospitalization: 2 cases of *P. falciparum* (case nos. 7 and 23), 2 cases of *P. vivax* (case nos. 10 and 42), 1 case of *P. knowlesi* (case no. 47), and 1 case of *P. malariae*-*P. falciparum* mixed infection (case no. 14). In all cases, according to the progress of recovery from malaria, daily M scattergrams visually showed the density of the clusters of *Plasmodium* infected RBCs to become thinner day by day (LM mode: Fig. 6a, PD mode: Additional file 5). The parasitaemia counted by microscopy and the MI-RBC% detected by both the LM and PD modes of the XN-31p were compared. Note that parasite counting under microscopic observation included damaged or dead parasites affected by drugs, such as pyknotic forms caused by artemether treatment.

Changes of parasitaemia until parasite clearance as determined by microscopic observation and the XN-31p were well concordant (LM mode: Fig. 6b, PD mode: Additional file 5). For 3 cases (case nos. 7, 10, and 47), the days of parasite clearance determined by microscopic observation and the XN-31p (the LM and PD modes) matched completely. For 1 *P. falciparum* case (case no. 23), a small number of parasites were still detected by the LM and PD modes of the XN-31p on day 4 when parasite clearance was declared based on microscopic observation, and the patient was discharged. Further monitoring with the XN-31p was not feasible. For the *P. malariae*–*P. falciparum* mixed infection (case no. 14), the day of parasite clearance confirmed by microscopy was 2 days after the day when parasites were no longer detected by the LM and PD modes of the XN-31p. For 1 *P. vivax* case (case no. 42), monitoring was terminated before confirmation of parasite clearance both by microscopy and the XN-31p because the patient was discharged. In this case, on day 2, only gametocytes were detected by the microscopic observation at 0.014% parasitaemia, and the M scattergram showed a green-coloured cluster of parasites only, which corresponded to the gametocytes.

## Discussion

The present study using the XN-31p targeted cases of imported malaria in Japan, which the patients had acquired malaria through travel to endemic countries. The excellent performance of the XN-31p was revealed not only for the detection of *P. falciparum* cases but also for non-falciparum malaria cases. This is the first report of the measurement of *P. malariae* or *P. knowlesi* infection by the XN-31p.

High concordance of the MI-RBC% detected by the LM and PD modes of the XN-31p with the % parasitaemia value obtained by microscopy was observed regardless of the species of malaria parasites. This capacity of the XN-31p to produce the accurate % parasitaemia is of utmost importance for starting proper treatment of falciparum malaria patients. In fact, conventional microscopy which can provide us with the quantitative information is to be only performed by well-trained microscopists, which is a big challenge in hospitals in non-endemic regions. The XN-31 with less laborious procedures could overcome this issue.

Moreover, the sensitivity and specificity of the XN-31p was almost the same as that of microscopic observation performed by expert microscopists using thin blood smears. In fact, some of the health personnel in the endemic settings are performing the microscopic tests with only thick blood films. However, it is reported previously that the sensitivity and specificity of the XN-31p were almost identical to those of microscopic tests with thick blood films [11].

Reproducibility of the test results by the XN-31p was also confirmed by applying 10 repetitive measurements. It was notable that the results of the PD mode, in which blood diluted 7 times is measured, were almost the same as those of the LM mode, which uses whole blood, suggesting that an amount as small as 20 µL of whole blood would be sufficient for the XN-31p to produce reliable test results.

The general detection limit for malaria parasites by microscopic observation was reported to be 50 parasites/µL of blood [4]. However, as defined in the product profile by the Sysmex Corporation, the LoQ (limit of quantitation) of the XN-31p are 20 parasites/µL of blood (by the LM mode) and 40 parasites/µL of blood (by the PD mode). One case in the present study is illustrative of the sensitivity of the XN-31p. The patient was a Ghanaian male who was microscopically positive with only 1 *P. falciparum* parasite found in 360,000 RBCs as counted by one of the authors, which was calculated to be 10–16 parasites/µL. This case was detected as malaria positive by the LM mode, with a flagging message of “Malaria?(UNC)”, but negative by the PD mode. Thus, the LoD (limit of detection) of the LM mode of the XN-31p could be much lower than the LoQ value defined by the company. This result is consistent with a recent report [12] that estimated LoD and LoQ values for the LM mode of the XN-31 using cultured *P. falciparum*. Recently, asymptomatic malaria cases with low-density parasitaemia have been discussed as potential reservoirs for transmission in endemic areas [16–18]. Nucleic acid tests (NATs), such as PCR [6–8] or LAMP (loop-mediated isothermal amplification) [19], which are sensitive enough to detect 1–10 parasites/µL of blood, are believed to be more adequate for the surveillance of these asymptomatic parasite carriers. Now, however, the XN-31p might also be considered as a tool powerful enough to elucidate these hidden malaria groups.

In the present study, 2 samples were suggested to show abnormal patterns of M scattergrams by the flagging message of “MI-RBC Abn Scattergram”. In previous studies using the XN-30, it was pointed out that such abnormal patterns were related to the health conditions of the participants. For example, infants or malnutrition of the participants was related to the appearance of such abnormal patterns [9]. The existence of reticulocytes, which may not have been correctly identified, might contribute to such abnormal patterns [10]. A recent study also showed that various RBC abnormalities cause “MI-RBC Abn Scattergram” and that these abnormalities sometimes even cause exact false-positive results by the XN-31 [12]. These patterns should be treated carefully and examined with other methods at clinical sites, and more examination is needed in future studies.

In most of the present cases, the flagging messages suggested by the XN-31p well differentiated the *P. falciparum* and non- *P. falciparum* cases. In the case of the *P. falciparum*-*P. ovale* mixed infection, the flagging message given by the XN-31p was “Malaria?(P.f)”, but the existence of non- *P. falciparum* parasite infected RBCs could be predicted by the M scattergram as a small sub-cluster appearing beside the main *P. falciparum* clusters.

In the case of *P. knowlesi* infection, the flagging message was also “Malaria?(P.f)”, but the location of the cluster of parasite infected RBCs in the M scattergram was slightly different from the typical *P. falciparum* patterns, suggesting the possibility of infection by some other *Plasmodium* species. In this case, the negative RDT result strongly supported the prediction, and microscopic observation and the nested PCR verified this case to be a *P. knowlesi* infection. Imported cases of *P. knowlesi* are quite rare in Japan, and this case was the 2nd reported case [20].

In the case of *P. malariae* infection, the shape of the cluster shown in the M scattergram was different from that of *P. falciparum* or *P. vivax*. Although it is known that the trophozoites of *P. malariae* and *P. knowlesi* found in the microscopic observations of thin-blood smears are similar by showing a “band form” [2, 3, 21], the patterns of *P. malariae* and *P. knowlesi* were absolutely different in the M scattergram of the XN-31p. Such characteristics are expected to be better reflected in the algorithm of the XN-31p, and detailed differentiation of *Plasmodium* species may be possible in the near future.

The XN-31p also detected non-ring-stage parasites of *P. falciparum*, such as schizonts and gametocytes. The appearance of schizonts in the peripheral blood of patients with *P. falciparum* infections is an important sign of severe malaria [15]. However, gametocytes are sexually developed stage parasites that are transmissible to mosquitoes, but they do not cause malaria symptoms [22]. Such ability of the XN-31p to distinguish parasite stages could be helpful in understanding a patient’s clinical condition. From the M scattergrams produced by the XN-31p measurements, more detailed developmental stages of the parasites might be predicted. Because the blood cells are treated by the Lysercell M automatically in the XN-31p before laser detection, and the parasite-infected cells are shrunken to the size of the parasite cell inside the host cells [14]. Thus, the FSC data reflects the size of the parasite cells inside RBCs, so that it was possible to guess the detailed developmental stages with the combination of the SFL data. Using the FSC information given by the XN-31p, could even differentiate the young ring-stage parasites, which are suspected to have immediately invaded the RBCs, and old ring-stage parasites, which are suspected to just become mature trophozoites. For example, in the M scattergrams of the 2 *P. vivax* cases, both included the red-coloured cluster indicative of ring stages (Fig. 3d, e). The vertical positions of the red cluster in these cases were different from each other, suggesting that the average cell size of the ring-stage parasites in case No. 42 was larger than that in case No. 10. Such information can possibly help to predict the course of parasite proliferation in a patient’s body and can lead to an understanding of the progression of the disease condition and the therapeutic effect (Fig. 6a, b).

Microscopy has been the gold standard for the diagnosis of malaria, and much information can be obtained by observation under the microscope, such as the number of parasites, characteristic morphology of *Plasmodium* species, developmental stages, and effects of malaria treatments. However, a number of alternative methods for malaria diagnosis have now been developed whose principle of operation is to detect some substance derived from malaria parasite. For example, the RDT detects parasite-derived antigens in the patient’s blood, and NATs, as represented by PCR and LAMP, detect DNA fragments derived from the parasites. However, the XN-31p measurement is the first diagnostic method other than microscopy to provide information on parasite morphology that suggests the clinical condition of the patient.

In the course of monitoring malaria treatment, the XN-31p could detect decreasing parasitaemia, which suggested the usefulness of the XN-31p in the evaluation of malaria treatment. However, it was difficult to differentiate normal parasite forms from damaged or dead parasite forms in the M scattergrams. A previous study reported the use of the XN-30 in the screening of malaria drug candidates [23]. In that study, the M scattergrams indicated which developmental stage of the cultured *P. falciparum* was affected by each anti-malarial drug, and parasite growth inhibition could be shown visually. Nevertheless, differentiation of dead and alive parasites at the same developmental stage was difficult. Thus, careful attention must be paid when using the XN-31p for the evaluation of treatment. On the other hand, the parasite clearance time indicated by the XN-31p and that by microscopic observation were almost the same, with only a one-day difference. Data obtained by the XN-31p following treatment could strongly support the confirmation of parasite clearance.

## Conclusion

The XN-31p could provide instructive information on the MI-RBC and flagging messages regarding the *Plasmodium* species, with excellent sensitivity and specificity which were much better than the RDT, in fact, showing a good correlation with the nested PCR and the microscopic observation. The results were also obtained in one minute and, thus, the procedure was far less laborious and the time required for the tests was overwhelmingly shorter than microscopy or the PCR. Moreover, the XN-31p did not require such trained skills as were indispensable for the PCR testing or microscopy. This newly developed haematology analyzer will be useful for aiding in the clinical diagnosis of malaria not only in endemic areas but also in non-endemic countries where suspected malaria cases are rare and healthcare professionals do not have sufficient skills to diagnose patients with imported malaria.

In addition, the M scattergrams produced by the XN-31 could also suggest information regarding characteristic morphology of both *Plasmodium* species and developmental stages that can be potentially helpful in predicting the course of parasite proliferation in a patient’s body leading to an understanding of the progression of the disease and the therapeutic effect.

## Supplementary Information

**Additional file 1. **A flowchart illustrating the counting manner in the microscopic observations.

**Additional file 2. **Detailed profile of all patients recruited to this study.

**Additional file 3. **Comparison of the results of the nested PCR and microscopy or the XN-31p for evaluation of the ability of each method to discriminate *Plasmodium* species.

**Additional file 4. **All MI-RBC# and MI-RBC% data of the 10 repetitive measurements of 5 samples obtained by both the LM and PD modes of the XN-31p.

**Additional file 5. **Monitoring of the cases by the XN-31p (PD mode) during the treatment of malaria. **a** The M scattergrams obtained by the PD mode from daily measurements. **b** Changes in parasitaemia in each case during the monitoring period. The vertical axes show parasitaemia detected by microscopy (dashed lines) and PD (solid lines) mode. The black arrows indicate the days when parasite clearance in the patients’ blood was declared by microscopy, and the gray arrows indicate those detected by PD mode. Pf: *P. falciparum*, Pv: *P. vivax*, Pm: *P. malariae*, Pk: *P. knowlesi*.

## Data Availability

The dataset generated during this study is included in this published article and its additional files.

## Abbreviations

RBC: Red blood cell
CBC: Complete blood count
NCGM: National Center for Global Health and Medicine
RDT: Rapid diagnostic test
FSC: Forward-scattered light
SFL: Side fluorescent light
MI-RBC: Malaria-infected red blood cell
LM: Low malaria
PD: Pre-dilution
UNC: Unclassified
Abn: Abnormal scattergram
PPV: Positive predictive value
NPV: Negative predictive value
NAT: Nucleic acid testing
