# Supplementary figures and images for: Clinical performance testing of the automated haematology analyzer XN-31 prototype using whole blood samples from patients with imported malaria in Japan
Source: Malar J. 2022 Jul 30;21:229. doi: 10.1186/s12936-022-04247-x (PMC9338637; doi:10.1186/s12936-022-04247-x)

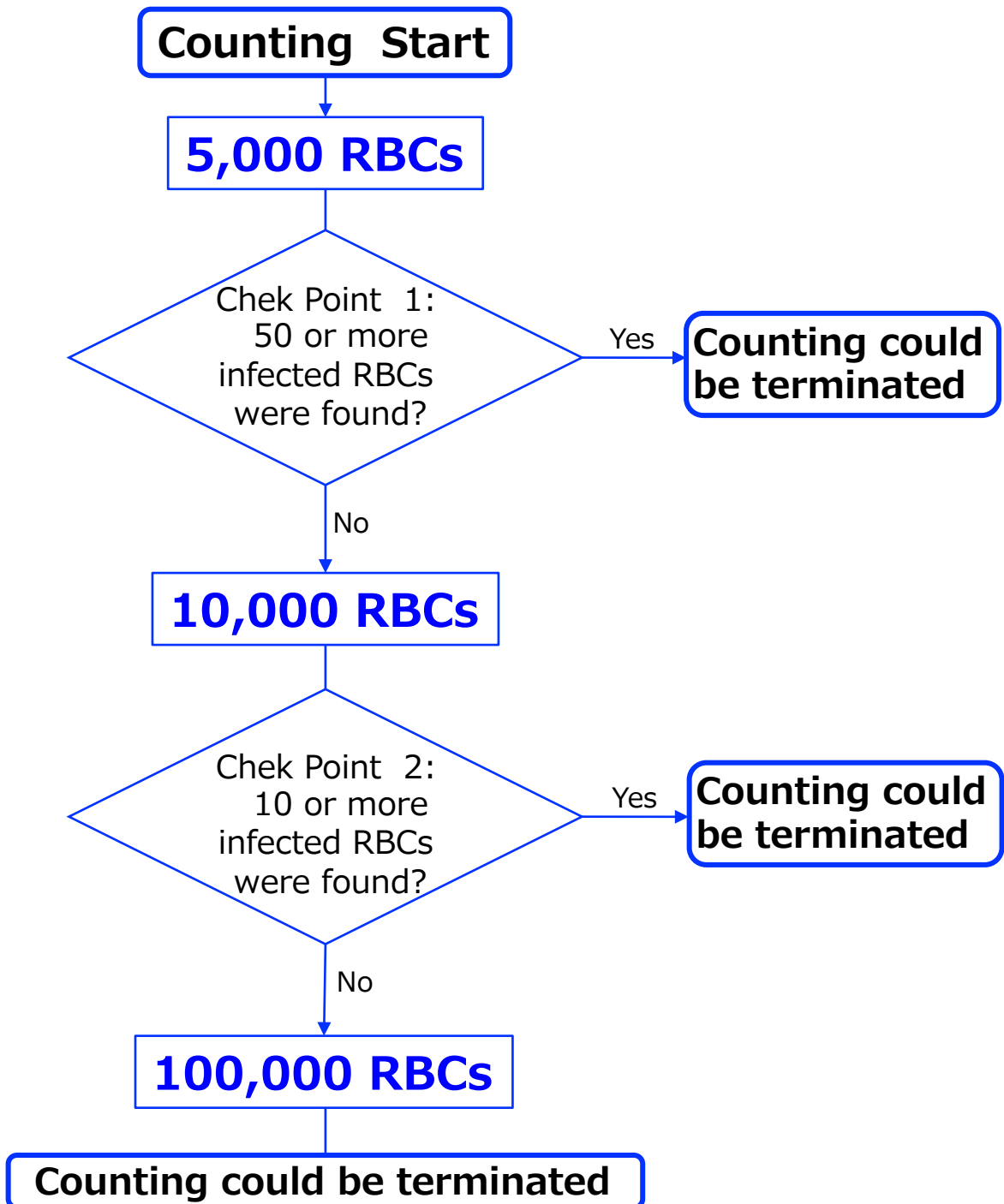

Supplement: Supplementary file 1 — Additional file 1. A flowchart illustrating the counting manner in the microscopic observations. [file 12936_2022_4247_MOESM1_ESM.pdf]
