# Supplementary material for: Clinical performance testing of the automated haematology analyzer XN-31 prototype using whole blood samples from patients with imported malaria in Japan
Source: Malar J. 2022 Jul 30;21:229. doi: 10.1186/s12936-022-04247-x (PMC9338637; doi:10.1186/s12936-022-04247-x)

Case 7 Pf

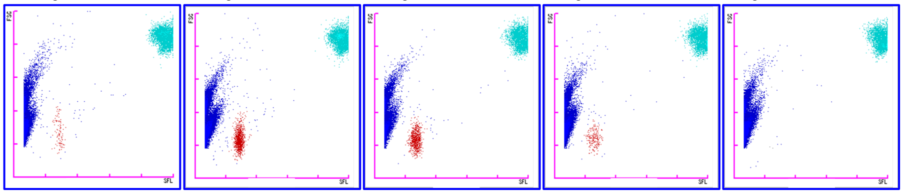

	Day 1	Day 2	Day 3	Day 4	Day 5
PD mode	0.0033	0.0367	0.0248	0.0072	Negative
Microscopy	0.0015	0.034	0.019	0.0015	0

Case 10 Pv

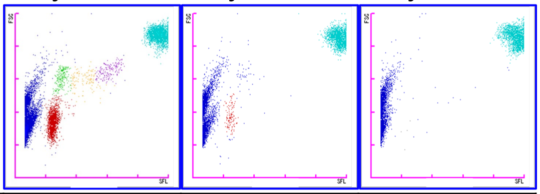

	Day 1	Day 2	Day 3
PD mode	0.0614	0.0040	Negative
Microscopy	0.083	0.0054	0

Case 47 Pk

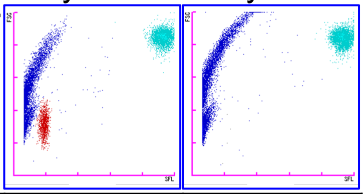

	Day 1	Day 2
PD mode	0.0421	Negative
Microscopy	0.062	0

Case 23 Pf

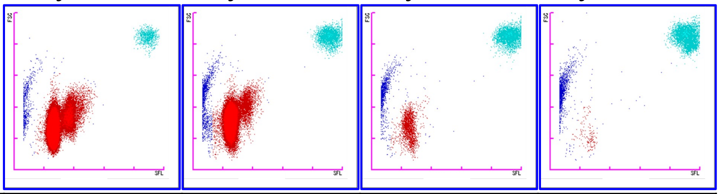

	Day 1	Day 2	Day 3	Day 4
PD mode	8.4209	3.1347	0.0714	0.0046
Microscopy	9.34	3.14	0.036	0

Case 42 Pv

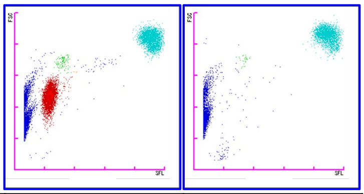

	Day 1	Day 2
PD mode	0.1954	0.0010
Microscopy	0.255	0.0014

Case 14 Pm-Pf

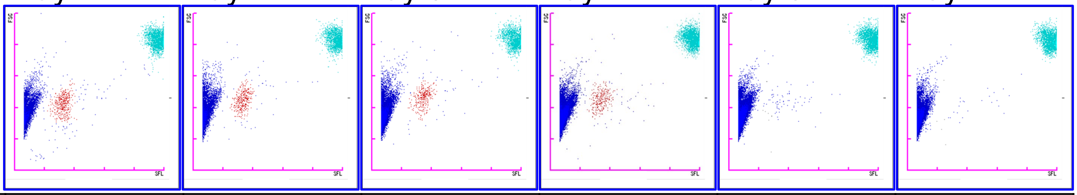

	Day 1	Day 2	Day 3	Day 4	Day 5	Day 7
PD mode	0.0202	0.0183	0.0145	0.0099	Negative	Negative
Microscopy	0.021	0.024	0.018	0.014	0.0043	0

b

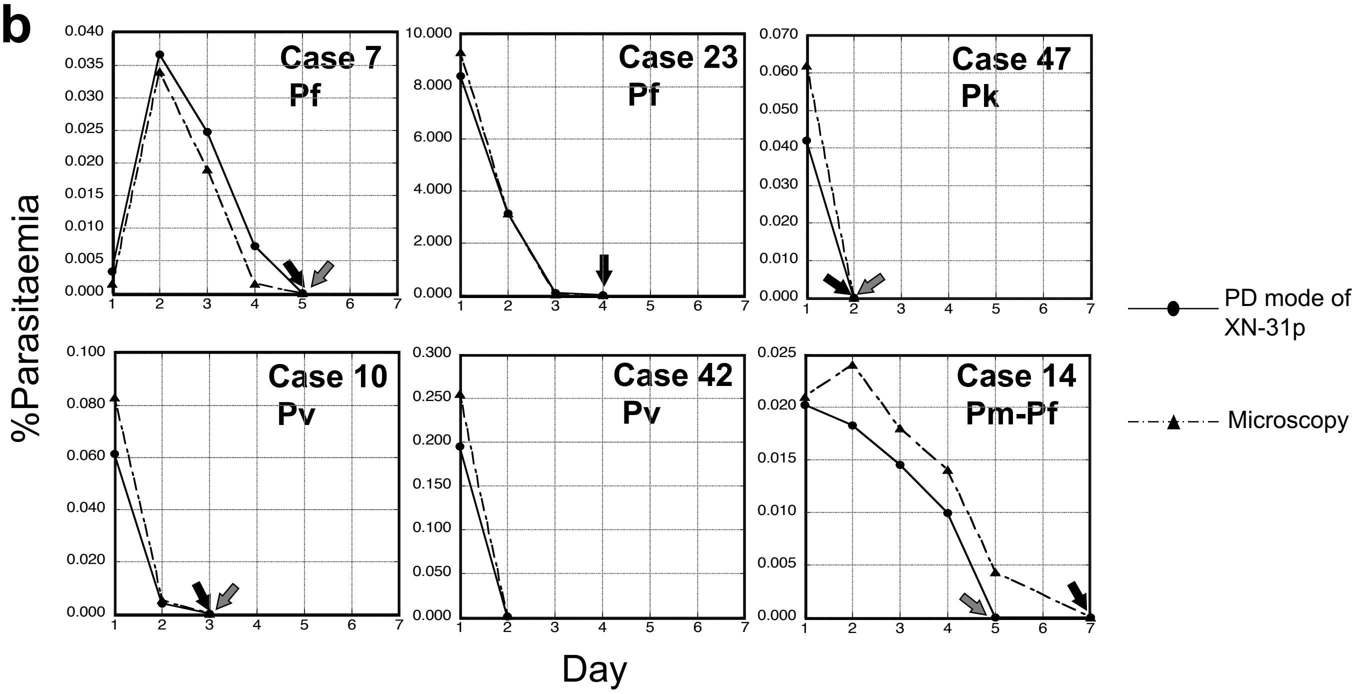

Supplement: Supplementary file 5 — Additional file 5. Monitoring of the cases by the XN-31p (PD mode) during the treatment of malaria. a The M scattergrams obtained by the PD mode from daily measurements. b Changes in parasitaemia in each case during the monitoring period. The vertical axes show parasitaemia detected by microscopy (dashed lines) and PD (solid lines) mode. The black arrows indicate the days when parasite clearance in the patients’ blood was declared by microscopy, and the gray arrows indicate those detected by PD mode. Pf: P. falciparum, Pv: P. vivax, Pm: P. malariae, Pk: P. knowlesi. [file 12936_2022_4247_MOESM5_ESM.pdf]
